# Synthesis and Characterization of Elongated-Shaped Silver Nanoparticles as a Biocompatible Anisotropic SERS Probe for Intracellular Imaging: Theoretical Modeling and Experimental Verification

**DOI:** 10.3390/nano9020256

**Published:** 2019-02-13

**Authors:** Carlos Caro, Pedro Quaresma, Eulália Pereira, Jaime Franco, Manuel Pernia Leal, Maria Luisa García-Martín, Jose Luis Royo, Jose Maria Oliva-Montero, Patrick Jacques Merkling, Ana Paula Zaderenko, David Pozo, Ricardo Franco

**Affiliations:** 1 Department of Physical, Chemical and Natural Systems, Universidad Pablo de Olavide, Carretera de Utrera Km 1, 41013 Seville, Spain; josolimon@gmail.com (J.M.O.-M.); pjmerx@upo.es (P.J.M.); apzadpar@upo.es (A.P.Z.); 2 Departamento de Química, UCIBIO, REQUIMTE, Faculdade de Ciências, Universidade NOVA de Lisboa, 2829-516 Caparica, Portugal; 3 CABIMER, Andalusian Center for Molecular Biology and Regenerative Medicine, Av. Americo Vespucio, 24, 41092 Sevilla, Spain; jaime.munoz@cabimer.es; 4 BIONAND, Andalusian Centre for Nanomedicine and Biotechnology, Junta de Andalucía, Universidad de Málaga, 29590 Málaga, Spain; mpernia@us.es (M.P.L.); mlgarcia@bionand.es (M.L.G.-M.); 5 Departamento de Química e Bioquímica, LAQV-REQUIMTE, Faculdade de Ciências, Universidade do Porto, 4169-007 Porto, Portugal; pedro.cq1@gmail.com (P.Q.); efpereir@fc.up.pt (E.P.); 6 Department of Medical Biochemistry, Molecular Biology and Immunology, Universidad de Sevilla, Av. Sanchez Pizjuan, 4, 41009 Sevilla, Spain; 7 Department of Organic and Pharmaceutical Chemistry, Universidad de Sevilla, 41012 Seville, Spain; 8 Department of Biochemistry, Molecular Biology and Immunology, Universidad de Málaga, 29071 Málaga, Spain; joseluisroyo@uma.es

**Keywords:** surface enhanced Raman scattering, SERS, finite element method, density functional theory calculations, cell labeling, cancer

## Abstract

Progress in the field of biocompatible SERS nanoparticles has promising prospects for biomedical applications. In this work, we have developed a biocompatible Raman probe by combining anisotropic silver nanoparticles with the dye rhodamine 6G followed by subsequent coating with bovine serum albumin. This nanosystem presents strong SERS capabilities in the near infrared (NIR) with a very high (2.7 × 10^7^) analytical enhancement factor. Theoretical calculations reveal the effects of the electromagnetic and chemical mechanisms in the observed SERS effect for this nanosystem. Finite element method (FEM) calculations showed a considerable near field enhancement in NIR. Using density functional quantum chemical calculations, the chemical enhancement mechanism of rhodamine 6G by interaction with the nanoparticles was probed, allowing us to calculate spectra that closely reproduce the experimental results. The nanosystem was tested in cell culture experiments, showing cell internalization and also proving to be completely biocompatible, as no cell death was observed. Using a NIR laser, SERS signals could be detected even from inside cells, proving the applicability of this nanosystem as a biocompatible SERS probe.

## 1. Introduction

In recent years, Raman spectroscopy has been studied extensively, given its wide range of applications especially those based on the Surface Enhanced Raman Scattering (SERS) effect [1,2,3,4,5,6,7,8], by which Raman-active compounds can be detected at very low concentrations. This approach is especially valuable since Raman scattering is a very inefficient spectroscopic process and its use for detection purposes is limited. In SERS, to obtain an efficient amplification, the organic compound should be near the surface of metallic nanoparticles (NPs), especially those based on the reduced form of metals such as copper, gold or silver, where the Raman signals can be amplified by factors as high as 10^14^–10^15^ [9,10,11]. In this manner, the ultimate detection limit could be a single analyte molecule [12,13,14]. The net SERS effect is the outcome of two mechanisms, which can act simultaneously or separately. These have come to be called electromagnetic (EM) and chemical (CM), respectively [15,16]. The EM is a consequence of the local increase of electric field in the vicinity of the NPs, due to surface plasmon excitation. This increase leads to a more intense absorption of electromagnetic radiation in molecules in the vicinity of the NPs, and therefore to enhanced Raman scattering [17]. The CM arises from changes in the polarizability of a given molecule, owing to charge transfer interaction between electronic states of the molecule and the NPs surface, leading also to increased Raman signals [18]. The CM depends exclusively on the Raman-active molecule and its interaction with the surface of NPs, while the EM depends on the surface plasmon resonance (SPR) [19,20,21]. SPR can be defined as a quantized collective oscillation of electrons confined within a metal/dielectric boundary that strongly interact with electromagnetic fields. According to the latest findings, Raman spectroscopy parallel to the SERS effect can be considered as one of the most powerful techniques currently available for sensing applications [22,23,24,25,26,27].

Rhodamine 6G (R6G) is a rigid molecule composed of a xanthene ring, an ethoxycarbonylphenyl group, and two ethylamino groups. Rhodamine 6G exhibits a high Raman cross-section and has been extensively studied experimentally, and so it represents a suitable benchmark molecule for SERS studies. Specifically, R6G has been shown to exhibit SERS effect on films composed of aggregates of thiol-immobilized silver-based NPs (AgNPs) [28]. In this sense, it is worth to mention that detailed data analysis from computational studies have been useful and valuable to understand SERS experimental-based phenomena, including the vibrational analysis of the R6G cation [29,30,31], the SERS effect on AgNPs to characterize the highest occupied molecular orbital (HOMO) and lowest unoccupied molecular orbital (LUMO) states [32], the effect of geometry on SERS of minimal silver clusters [33], as well as the spectroscopic states involved in the transition in resonance Raman [34].

Since SERS is able to detect Raman-active compounds at picomolar concentration, it could be used as a powerful tool for diagnosis and/or monitoring disease progression through tracking of specific biomarkers [35]. Typically, NPs used as SERS nanotags are obtained by interaction or anchoring of a Raman dye onto the surface of metallic NPs such as gold- or silver-based NPs. Subsequently, these NPs-dye conjugates are coated with a biocompatible, protective layer such as polymers or proteins [36,37]. For instance, gold nanoparticles (GNPs) conjugated with bovine serum albumin (BSA) have been used with encouraging results for *in vivo* multiplexing SERS [38].

Development of new non-spherical nanoparticles is becoming an option to increase SERS amplification for intracellular applications [39], since this type of anisotropic nanoparticles produce a high confinement of electromagnetic energy at sharp-nanostructured corners and edges [40]. Among them, of particular interest are star-shaped [6,41] or elongated/rod-shaped nanoparticles [42] which show considerable field amplifications near the tips, i.e., at the extremities of the particle long axis. In addition, another advantage of these nanoparticles is the possibility of using lasers in the near-IR, due to a shifting of the SPR, which is a particularly interesting feature for their usage in biological systems. Thus, an evaluation of the suitability of these nanoparticles for SERS applications can be made by using the finite element method to determine the near field around the particle, followed by comparison of the intensity of the electromagnetic field enhancement for different nanoparticle shapes and laser wavelengths [43,44]. In this sense, it has been shown that in general AgNPs have an intrinsically higher enhanced factor (EF) compared to GNPs [45]. However, Yuan et al. [46] demonstrated that EF of gold nanostars and silver spheres are similar, and both are considerably higher than gold spheres, making those particular NPs suitable for cellular SERS labelling. Remarkably, recent studies have been reported the use of AgNPs in cellular SERS labelling [39,47,48], suggesting that these could discriminate between different cell compartments for *in vitro* experimental approaches.

Nevertheless, for *in vivo* purposes, SERS-based nanoparticles should be compatible with the use of lasers with wavelengths within the so-called biological window, in order to minimize the absorption from living tissues [49,50].

Based on the above, the aim of this work was to obtain a biocompatible nanosystem that allows sensitive intracellular SERS probing. For that purpose, anisotropic AgNPs with an elongated shape were conjugated with the R6G Raman active dye and BSA. These elongated shape AgNPs show a SPR band in the near-IR region. The BSA coating entraps the Raman active dye and confers colloidal stability as well as biocompatibility to the nanosystem. To the best of our knowledge, this is the first time that analytical enhanced factor (AEF) is analyzed both by density functional theory (DFT) and finite element method (FEM) calculations. Remarkably, our results are in good agreement with SERS experimental findings. In this sense, FEM calculations show a significant field enhancement at 785 nm, making the elongated AgNPs excellent SERS probes. Finally, we were able to label by intracellular SERS a carcinoma cell line (A431), using a laser in the near-IR region and without any detectable toxicity to cells.

## 2. Materials and Methods 

### 2.1. Materials

All chemicals were of reagent grade and have been used without further purification: tetraethylrhodamine hydrochloride (Rhodamine 6G) and BSA from Sigma Aldrich (Madrid, Spain), silver nitrate, sodium citrate, ascorbic acid and hydroxylamine hydrochloride from Panreac (Barcelona, Spain). Water was purified using a Milli-Q (18.2 MΩ) water system from Millipore (Madrid, Spain).

### 2.2. Synthesis of Silver Nanoparticles (AgNPs)

A solution containing silver nitrate (3.06 mM) and sodium citrate (6.2 mM) was prepared in 25 mL of Milli-Q water in a round bottom flask dipped in an ice bath and was stirred at 700 rpm. Upon complete dissolution of both salts, 0.5 mL of a 56.7 mM solution of ascorbic acid was quickly added and the reaction was allowed to proceed for additional 5 min. After this, the dispersion was centrifuged at 4000 rpm for 20 min and the resulting pellet was redispersed in Milli-Q water. This process was repeated 3 times to remove unreacted reagents, resulting in a final dispersion of AgNPs.

### 2.3. Transmission Electron Microscopy (TEM)

TEM measurements were performed in a 200 kV Philips CM-200 microscope (Philips, Amsterdam, Netherlands) with a supertwin objective lens, a LaB6 filament and side-entry specimen holder (point resolution 0.24 nm). To prepare the TEM samples, ~5 µL of solution containing the sample were removed from the bottom of the vial, followed by the removal of ~5 µL of the supernatant into the same pipette tip. Two drops of this suspension were deposited on a carbon-coated copper TEM grid (Ted Pella, Redding, CA, USA). The instrument is equipped with an energy-dispersive X-ray spectroscopy (EDX) detector (EDAX Inc., Tilburg, Netherlands). Signals from the elements Si, Cu and C are originated from carbon-coated copper TEM grid. Taking into consideration that EDX is based on the principle that each element has a unique atomic structure (with a unique set of peaks on its electromagnetic emission spectrum), we can discriminate between silver NPs and the TEM grid. The size histogram was prepared using Image-Pro Plus software (Media Cybernetics, Rockville, MD, USA), based on the measurements obtained from more the 150 nanoparticles.

### 2.4. Preparation of AgNPs@R6G@BSA

AgNPs concentration was determined by direct weighing of a sample after drying. A volume of 0.65 mL of the AgNPs suspension (2.84 mg/mL) was incubated with 120 µL of R6G 10^−3^ M. After two hours of incubation, 2 mL of a 50 µM BSA solution in phosphate buffer saline (PBS) was added and allowed to incubate overnight, followed by centrifugation at 4000 rpm for 30 min and re-suspension in Milli-Q water. The amount of bound R6G was determined using the UV-Vis spectrum (Ocean Optic, Largo, FL, USA) of a R6G blank solution (mixing 120 µL R6G and 0.65 mL of Milli-Q water) as a standard and comparing with the supernatant obtained after incubation with the particles and centrifugation. The remaining concentration of R6G in AgNPs was calculated to be 9.8 × 10^−7^ M (4.1 × 10^−10^mol R6G/mg AgNPs).

### 2.5. UV-VisSpectroscopy

The UV-Vis spectra were recorded with an Ocean Optic spectrometer (Ocean Optic, Largo, FL, USA) equipped with a HR4000 detector with a quartz tray with a light path of 1 cm. 

### 2.6. Dynamic Light Scattering (DLS)

The size distribution measurements of the nanoparticles were performed on a Zetasizer Nano ZS90 (Malvern, UK). The nanoparticles were dispersed in milli-Q water or PBS at a concentration of 50 mg/L of Ag. The measurements were done on a cell type: ZEN0118-low volume disposable sizing cuvette, with 173° Backscatter (NIBS default) as angle of detection. The measurement duration was set to automatic and three measurement repeats. The analysis model was set to general purpose (normal resolution). The polydispersity index (PDI) is determined using a Malvern software (Malvern, UK), based on a cumulants analysis that performs a single fit to the correlogram. 

### 2.7. Raman Spectroscopy

Raman IR spectra were measured on a Bruker Senterra Confocal Raman Microscope (Bruker, Hamburg, Germany) equipped with a 785 nm Ne laser and a DU420A-OE-152 detector (Oxford Instruments, Abingdon, UK). A 50× objective was used for all measurements, with a slit aperture fixed to 50 μm and an integration time of 100 s with a laser power of 50 mW. All spectra were recorded with a 3 cm^−1^ resolution. In order to calculate the analytical enhanced factor (AEF), measurements were carried out on a Labram 300 Jobin Yvon spectrometer (Horiba Jobin Yvon, Bensheim, Germany) equipped with a 17 mW HeNe laser operating at 632.8 nm and with an air-cooled CCD detector. Spectra were recorded as extended scans. The laser beam was focused with a 50× Olympus objective lens (Olympus, Tokyo, Japan). The laser power at the surface of the samples was fixed with the aid of a neutral density filter (optical density 0.3). All measurements were performed using 5 scans with 25 s of laser exposure.

### 2.8. Density Functional Theory (DFT) Calculations

DFT calculations were performed with the Gaussian 2009 program [51], using B3LYP exchange and correlation functional as a well-established and robust method along with DGDZVP as basis set (Gaussian, Wallingford, CT, USA). Structure optimizations of the R6G molecule and of the metal/R6G complex were carried out in vacuum with a very tight convergence criterion. All the presented spectra lacked imaginary frequencies, indicating that the minimization yielded true (at least local) minima. Calculated spectra were broadened by a 5 cm^−1^ convolution to facilitate comparison with experimental spectra. No shifting or scaling of wavenumbers was performed in any of the represented spectra. Raman spectra were obtained according to the methodology known from the literature [29], calculation of Raman intensities was based on Placzeks polarizability theory. It should be noted that the theoretical values were obtained within the double harmonic approximation, i.e., the force constants were assumed to be harmonic, and only the linear terms were retained in the series expansion of the polarizability tensor components with respect to a normal mode [51]. Spectra were calculated at room temperature and for a 785 nm wavelength. The basis set DGDZVP was applied to the silver-R6G conjugates. DGDZVP, as a full electron basis set, provides roughly 50% more basis functions than other basis set such as LANL2DZ and spectra obtained were found to compare more favorably in preliminary calculations. This has also been reported for related systems [52,53].Although it is customary to apply a scaling factor of 0.975 to all computed frequencies due to the finite size of the basis set employed, this has not been done in this work.

### 2.9. Finite Element Method (FEM) Calculations

FEM calculations were performed using a commercial software package, Comsol Multiphysics 4.4 (Comsol, Burlington, MA, USA) to obtain the near field enhancement around the nanoparticle at the wavelengths of the Raman lasers used (633 and 785 nm). A particle geometry similar to the most elongated structures visualized by TEM was simulated using an ellipsoid shape with dimensions of 30 × 13 × 5 nm. A single particle aligned along the *y*-axis of the simulation was used to calculate electric field intensities. The complex dielectric functions for silver were calculated from Johnson and Christy’s data [54]. Nanoparticles were enveloped in water simulated as an isotropic dielectric medium with a refractive index of 1.33. The meshing of the geometry was performed using a minimum element size of 1.2 nm and a total of 73,208 mesh elements. An incident electromagnetic field was applied along the *y*-axis direction and the simulation was solved using a direct solver for a scattered field formalism.

### 2.10. Cell Culture

Epidermoid (squamous cell) carcinoma cell line (A431) (from CLS Cell LinesServiceGmbH, Eppelheim, Germany) were cultured in Dulbecco’s Modified Eagle Medium (DMEM) supplemented with 4.5 g/L glucose, 2 mM L-glutamine, 10% fetal bovine serum (FBS) and 1% penicillin/streptomycin (ATCC, Manassas, VA, USA) at 37 °C in an incubator with a humidified atmosphere with 5% CO_2_ for 24 h at different experimental conditions.

### 2.11. Cytotoxicity Assays

Epidermoid A431 cells were seeded overnight in 96-well plates at a density of 10.000 cells per well, in a final volume of 200 μL. For 3-[4,5-dimethylthiazol-2-yl]-2,5-diphenyl tetrazolium bromide (MTT) and lactate dehydrogenase (LDH) determination, the medium was replaced the day after with fresh media containing AgNPs@R6G@BSA at concentrations ranging from 0.5 μg/mL to 140 μg/mL for 24 h. As a negative control, cells without exposure to NPs have been used while positive controls for cell death were obtained by exposing cells to 20% ethanol in the media culture.After treatment, the supernatant of each well was saved for LDH measurements and replaced by 100 μL of media containing the MTT labelling reagent (Roche Diagnostics GmbH, Mannheim, Germany) at a final concentration of 0.5 mg/mL. After further incubation for 4 h under a humidified atmosphere (37 °C, 5% CO_2_), 100 μL of the solubilization solution was added to each well. The plates were kept overnight in the incubator under a humidified atmosphere after complete solubilization of the formazan crystals. Then, spectrophotometric absorbances were measured using a microplate (ELISA) reader (Dynatech, Burlington, MA, USA) at 570 nm with 660 nm as the reference wavelength. LDH release after 24 h incubation with AgNPs@R6G@BSA was determined in the saved supernatant according to manufacturer’s instructions (Roche Diagnostics GmbH, Mannheim, Germany). Data were obtained from three independent experiments. Measurements were made in five replicas for each experimental condition.

The relative cell viability (%) and its error related to control wells were calculated by the equations:

(1) $$\mathrm{RelativeCellViability}\left(\mathrm{RCV}\right)\left(\%\right)=\left(\frac{\left[Abs\right]test-\left[Abs\right]Negative\;control}{\left[Abs\right]Negative\;control-\;\left[Abs\right]Positive\;control}\right)\times 100$$

(2) $$\mathrm{Error}\left(\%\right)=\mathrm{RCVtest}\times \sqrt{{\left(\frac{\left[\sigma \right]\mathrm{test}}{\left[\mathrm{Abs}\right]\mathrm{test}}\right)}^{2}+{\left(\frac{\left[\sigma \right]\mathrm{control}}{\left[\mathrm{Abs}\right]\mathrm{control}}\right)}^{2}}$$

Non-parametric tests were used for statistical analyses using IBM SPSS package v22 (SPSS, Chicago, IL, USA).

### 2.12. Flow Cytometry

For detection of apoptotic cells by flow cytometry, A431 cells were seeded overnight in 12-well plates at a density of 120,000 cells per well, in a final volume of 1 mL, and treated for 24 h at different nanoparticle concentrations. At the end of the experiment, the cells were fixed in an ice-cold solution of 70% (*v*/*v*) ethanol for at least 24 h, incubated for 10 min in DNA extraction buffer (0.2 MNa_2_HPO_4_, pH 7.8) and then incubated with 0.1% (*w*/*v*) RNAse (Thermofisher, Waltham, MA, USA) and 50 μg/mL propidium iodide at 37 °C for 30 min before analysis of the DNA content by flow cytometry using cell quest software (BD Biosciences, San Jose, CA, USA).

### 2.13. Dark Field Microscopy (DFM)

Epidermoid (squamous cell) carcinoma cell line (A431) (from CLS) were cultured on 1 mm thick and 1 cm in diameter (Goldseal number 1) glass coverslips for 48 h in Dulbecco’s Modified Eagle Medium (DMEM) supplemented with 4.5 g/L glucose, 2 mM L-glutamine, 10% fetal bovine serum (FBS) and 1% penicillin/streptomycin (ATCC) at 37 °C in an incubator with a humidified atmosphere with 5% CO_2_. AgNPs@R6G@BSA resuspended in DMEM (15 μg/mL final concentration) were added to the cell culture and kept under the same incubation conditions for 24 h. These assays were performed in duplicate. In order to ensure that only internalized nanoparticles are present in cell preparations, the cell culture was washed repeatedly with PBS 1×. After that, cells were fixed with paraformaldehyde and treated with Hoechst dye (dye with affinity for cell nuclei). Finally, the coverslips were mounted onto microscope slides using Vectashield Mounting Medium (Vector Laboratories, Burlingame, CA, USA) and analyzed using an upright fluorescence microscope Leica DM 2500 (Leica, Wetzlar, Germany), 100 W quartz halogen light and an external light source EL6000 for fluorescence images. Microscope was equipped with a dark field condenser without lens and a condenser upper lens DF D 0.80–0.95. Overlay fluorescence and DFM images were recorded using a Leica DFC 450 C camera and an HCX PL APO 100×/1.40–0.70 oil immersion objective.

## 3. Results and Discussion

**Figure 1:**
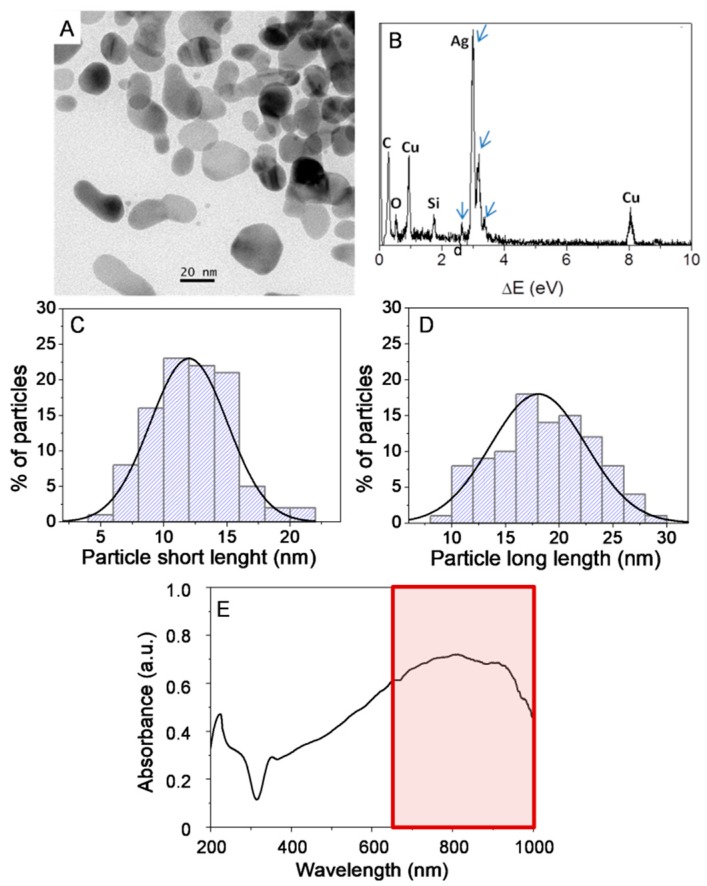
Microstructural characterization of AgNPs by TEM (**A**), EDX analysis of AgNPs (**B**), in which arrows indicate silver peaks. Particles short-length (**C**) and long-length (**D**) histograms-based on the measurements of over 150 nanoparticles. UV-Vis spectrum of AgNPs in solution (**E**) showing a maximum absorption in the NIR region (650–1350 nm in a red box). Signals from the elements Si, Cu and C are originated from carbon-coated copper TEM grid.

Anisotropic silver nanoparticles were easily produced by a one-pot method, using non-toxic reagents and water as solvent. The conditions of low temperature and mild reducing agent that were used favour a kinetic-driven process and thus the formation of anisotropic nanoparticles [55]. TEM images of the nanoparticles are shown in Figure 1. 

The TEM picture depicts a representative field in which the majority of particles exhibit an anisotropic and elongated irregular shape (Figure 1A). Energy-dispersive X-ray spectroscopy (EDX) results show a clear peak for Ag, Cu and C elements corresponding to the grid, while Si and O indicate are related to minor associated impurities (Figure 1B). The particle size distribution is shown in histograms (Figure 1C,D). The long-axis of the nanoparticles has an average distribution of lengths around 19 ± 5 nm, with more elongated populations reaching up to 30 nm, while the short-axis has dimensions of 13 ± 5 nm.

The UV-Vis spectrum of the AgNPs in solution shows a strong, broad SPR band with a maximum intensity in the range of 650–950 nm (Figure 1E). In addition, this SPR band makes these nanoparticles particularly suitable for studies in cells and tissues, due to the limited absorption of biological materials in the NIR region, which implies deeper penetration of radiation energy. In this sense, the laser wavelength used in this work (see details below) for *in vitro* experiments (785 nm) is within the high extinction range of the surface plasmon resonance of AgNPs, which favors the EM component of the SERS effect.

The particles have a high degree of stability, as DLS determinations in PBS (pH = 7.4) shown immediately after and one month after the synthesis procedure (Figure S1). Synthesized AgNPs showed a hydrodynamic diameter by DLS of 153.6 ± 0.9 nm. This high value might be explained by some degree of sample aggregation, especially because DLS is more sensitive to aggregates and large nanoparticles compared to small ones.

This partial sample aggregation is also reflected in the high value of the polydispersity index (PDI) of 0.184 ± 0.006. The large variety of shapes and sizes of the AgNPs could also contribute to this PDI value. After R6G physical adsorption procedure to AgNPs, the AgNP@R6G obtained showed an increase in size up to 269.2 ± 26 nm and due to some aggregation, the PDI was increased to 0.410 ± 0.098. In order to minimize this aggregation, BSA was used as a stabilization agent, yielding AgNP@R6G@BSA with smaller sizes (214.6 ± 0.5 nm) and PDI values (0.212 ± 0.007) compared to AgNP@R6G.

**Figure 2:**
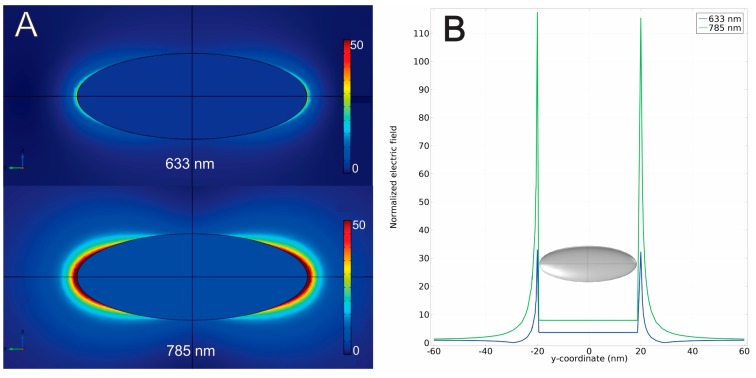
Local electric field enhancement (**A**) in the vicinity of one AgNP for incident radiation at 633 nm (**A**, top scheme) and at 785 nm (**A**, bottom scheme). Graphical representation of the electric field strength (**B**) (normalized to the incident field) along the *y*-axis of the simulation crossing the points of greater electric field intensity around the apexes of the AgNP.

The suitability of these AgNPs for SERS using a NIR laser was further confirmed by finite element method (FEM) calculations of the near field enhancement, assuming excitation lasers at 633 and 785 nm. FEM calculations were performed on a model particle of greater than average dimensions and with a high aspect ratio (shape similar to a rice grain) based on the results observed by TEM. This shape was chosen since the bigger and most elongated particles will have a greater contribution to the overall hotspots formed. The results from FEM calculations were mapped as color-coded field enhancements around the particle (Figure 2A). 

The numerical values for the calculated electric field strength were extracted along a line through the *y*-axis of the simulation that crosses the points of greatest electric field intensity around the apexes of the AgNPs. The electric field enhancement was then calculated by dividing these values by the incident electric field and was plotted against the distance on this line (Figure 2B). This allowed a numerical comparison to the maximum electric field enhancements for the two considered wavelengths. For the 633 nm laser, a moderate increase in the near field electromagnetic enhancement could be obtained, with a maximum of ca. 30-fold at a point near the particle surface.

However, for the 785 nm laser, we calculated a considerable field enhancement of 110–120-fold, and near points close to the surface of AgNPs corresponding to the apexes of a rice shape (Figure 2).

This considerable increase in the local field intensity stresses the potential usefulness of these particles as NIR capable Raman probes.

Once we have previously obtained an estimation of the EM component and in order to have a comprehensive understanding of our nanostructured system, we performed a computational study of R6G in Ag clusters to estimate the CM component of the SERS effect. 

**Figure 3:**
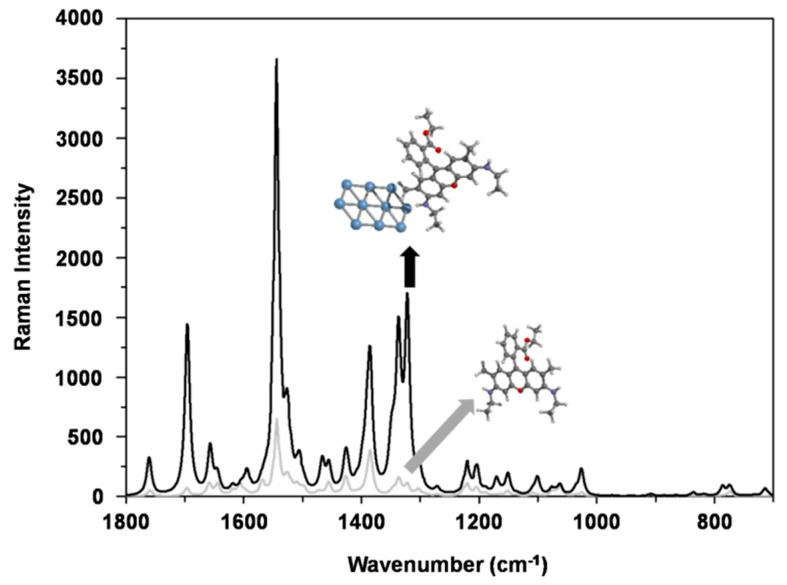
DFT calculation of R6G alone (grey line) and R6G interacting with a thin layer of 10 atoms of Ag (black line). The Ag layer causes a SERS effect leading to an increase in Raman signal intensity of one order of magnitude.

We chose a flat surface of 10 atoms of Ag to compare it with the computational Raman spectrum of isolated R6G. In Figure 3, we show the results from DFT calculations for the SERS effect on a flat surface composed of 10 atoms of Ag. 

Notably, the Raman intensity is 1 order of magnitude higher when compared to isolated R6G (area under the curve for the peak 1545 cm^−1^; calculated SERS = 429,898/isolated R6G = 39,682). These calculations demonstrate that Ag atoms themselves are very significant in SERS effect and are in good agreement with previous work modelling a flat surface of 6 atoms [56].

DFT calculations have some limitations, such as that calculated Raman spectra only take into account the interaction of the R6G with a single Ag atom of the thin layer. Actually, the nanoparticle has multiple Ag atoms interacting with the chemical compound, but the electronic structure of the nanoparticle is not considered in the previous estimation (Figure 3). Comparing computational enhanced and non-enhanced spectra, changes in the relative intensities of the signals are mainly in the lines appearing at 1695, 1335 and 1322 cm^−1^. These vibrations are primarily assigned to aromatic C–C stretching vibrations (Table S1) [57]. Another limitation using DFT to predict SERS spectra is that the calculation invariably gives rise to peaks that are not experimentally detected. Experimentally, some of the adjacent Raman lines in the SERS spectra will merge into a new and stronger line, and these adjacent lines will become difficult to distinguish due to limitations in instrumental resolution. In addition, a phenomenon that plays a dominant role in SERS enhancement, i.e., the local enhancement of the electromagnetic field due to the localized surface plasmon resonance (LSPR) is not taken into account in DFT [58]. Experimentally, the similarity of the spectra of pure R6G and AgNP@R6G@BSA (A and B in Figure S2) is useful for the identification of the adsorbed compound. The computational spectrum is similar to both experimental condensed phase spectra. This confirms that computational methods are in good agreement for this system (Figure S1C). It should be mentioned that all computationally-obtained vibrations have higher wavenumbers, as explained in the methods section. The peak positions and intensities are shown in Table S1. After adding the Raman indicator R6G to the AgNP solution, the next step in the synthesis was the incorporation of BSA to complete our design for the nanosystem.

This protein not only provides colloidal stability to the particles, but also prevents desorption of the Raman active dye and provides a biocompatible outer surface to the nanosystem. Furthermore, our AgNP@R6G@BSA platform opens the possibility for additional binding of antibodies which could ultimately lead to smart-targeted delivery approaches.

**Figure 4:**
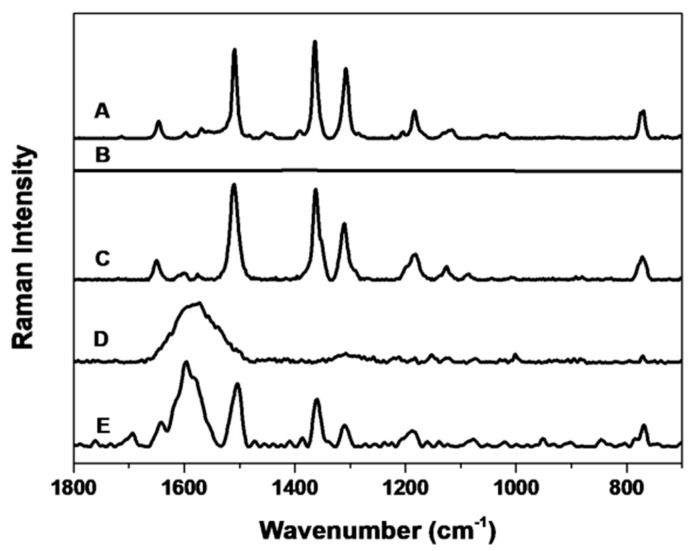
Raman and SERS spectra from different steps in the nanosystem synthesis. Raman spectra of pure R6G solution (**A**) and R6G at the same concentration used in the AgNP@R6G@BSA nanosystem (**B**). No spectrum is obtained from (**B**) because of the very low concentration of R6G. SERS spectra of AgNP@R6G (**C**), which is Raman active but has a very limited colloidal stability. AgNP@BSA is not Raman active (**D**). Raman spectra of AgNP@R6G@BSA nanosystem (**E**).

Pure solid R6G crystals and AgNP@R6G@BSA have very similar spectra (Figure 4A,E, respectively) indicating that BSA does not replace the R6G on the nanoparticle surface nor mask the R6G vibrations, allowing its detection by Raman. 

Furthermore, AgNP@R6G (Figure 4C) also has a very similar spectrum to pure R6G (Figure 4A) and to AgNP@R6G@BSA (Figure 4E). However, AgNP@R6G displays a very low colloidal stability in cell culture medium, and therefore it is not suitable as an *in vitro* Raman probe. In the spectrum of AgNP@R6G@BSA (Figure 4E), a broad line between 1550 and 1650 cm^−1^ can be assigned to BSA, since the spectrum of AgNP@BSA presents the same signal (Figure 4D). A hypothesis for the absence of changes in vibrations upon encapsulation with BSA could be that R6G interacts with the surface of the nanoparticle by one of the lateral amino groups, so that the main vibrations of xanthene and the aromatic ring are not affected. In Figure 4, the intensities of the spectra have been scaled for comparison. Moreover, it is noticeable that the R6G spectrum (Figure 4B), at the same concentration that the estimated for nanoparticles (AgNP@R6G and AgNP@R6G@BSA) shows no measurable signals.

A parameter which is widely used for a more accurate measurement of the SERS effect, other than just the Raman Intensity, is the AEF [59]. The AEF value can be defined by equation 1 in which the Raman intensity (*I*_SERS_) obtained at a particular concentration of analyte (*C*_SERS_) for the SERS experiment is related to the Raman intensity (*I*_NR_) for a concentration of analyte (*C*_NR_) obtained under normal conditions.

(3) $$\mathrm{AEF}=\frac{I_{\mathrm{SERS}}/C_{\mathrm{SERS}}}{I_{\mathrm{NR}}/C_{NR}}$$

All measurements for calculating the AEF were performed with a 633 nm laser since both the R6G control and AgNPs@R6G@BSA can be measured with the same time and laser power experimental conditions. Conversely, for the 785 nm laser measurement, the R6G control have to be measured under different acquisition conditions in comparison to the AgNP@R6G@BSA nanosystem, rendering non-comparable results and thus making it impossible to calculate a reliable AEF. The control Raman spectrum was measured on glass without silver nanoparticles, in a drop of R6G solution with a concentration of 10^−3^ M (data not shown). All intensities were calculated by using the area under the signal at 1360 cm^−1^, for both normal Raman intensity and SERS intensity. The AEF for the AgNP@R6G@BSA nanosystem was thus calculated as 2.7 × 10^7^. Although this is a remarkably high AEF, it should be noted that this calculation is done based on a Raman spectrum obtained with a 633 nm laser. As AgNPs show a higher near field enhancement as well as a much closer absorption maximum for the 785 nm wavelength than for the 633 nm (as shown in FEM calculations), it is possible that the AEF value for the 785 nm laser might be even higher. To estimate AEF for the 785 nm wavelength, we can first make an approximation to the EM contribution in this system for both lasers using the values for field enhancement obtained in the FEM simulations. The EM contribution to the SERS effect can be expressed, in a first approximation, by the fourth power of the ratio between the total electric field $E\left(r_{m},\nu \right)$ at the location of the molecule ($r_{m}$) and the incident field with E_inc_(ν) with ν being the frequency of the laser Equation (4) [60].

(4) $$EM_{SERS}\left(r_{m},\nu \right)={\left|\frac{E\left(r_{m},\nu \right)}{E_{inc}\left(\nu \right)}\right|}^{4}$$

Using the ratios between the total and incident electric field that was determined in the FEM calculations, we can estimate the magnitude of the EM component in our system. For the 633 nm laser and assuming the R6G to be near the spot with the highest near field enhancement, the EM contribution would be of the order of 8 × 10^5^. However, using equation 2 and the field enhancements obtained from the FEM calculations for the 785 nm laser under the same conditions, we can estimate that the EM contribution in this case could be as high as 2 × 10^8^. Therefore, the EM component for the 785 nm laser could be almost 3 orders of magnitude higher than at 633 nm, leading us to expect a much higher AEF at 785 nm. Regarding the EM component calculated for the 633 nm laser, this is a value consistent to the experimentally determined AEF of 2.7 × 10^7^ since the AEF depends on both the EM component and the CM contribution.

**Figure 5:**
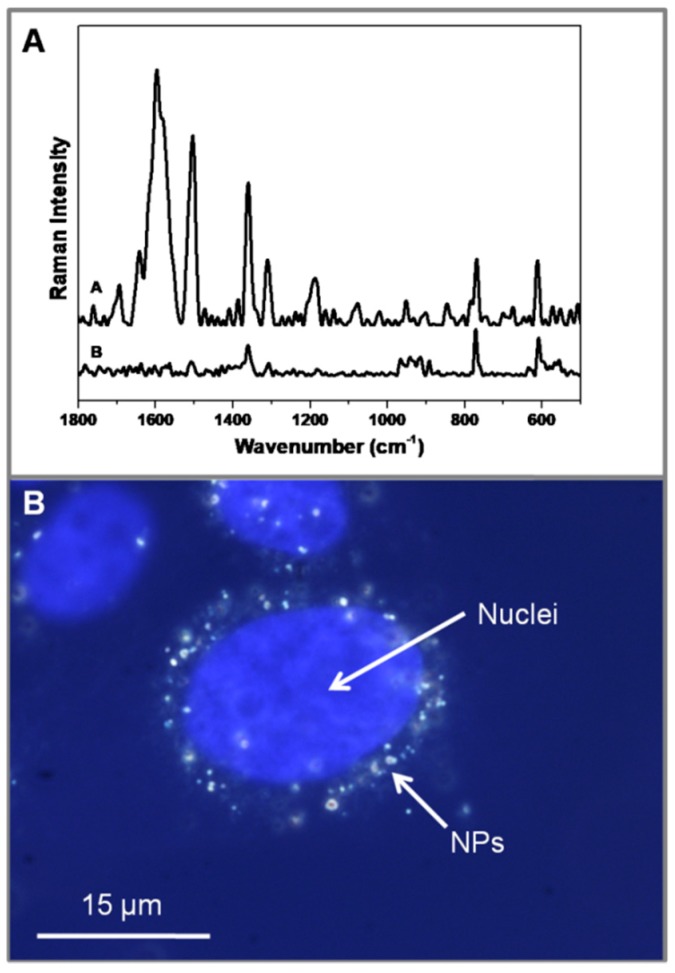
SERS spectra of AgNP@R6G@BSA (**A**. Inset A) and AgNP@R6G@BSA-treated human carcinoma cell line (A431) (**B**. Inset B). Overlay images of blue fluorescence channel (Hoechst dye labelling cell nuclei) and dark field microscopy (DFM) reveal nanoparticle internalization.

Finally, intracellular SERS measurements were performed on cells (A431 human carcinoma cell line), taking advantage of the very large SERS effect shown by the nanosystem even for extremely low levels of adsorbed R6G. For this purpose, we used a laser at 785 nm which takes advantage of the so-called biological window. Also, these AgNPs are particularly suited for use with this laser wavelength since the SPR maximum is in this region and the FEM calculations yielded a much higher near field enhancement for the 785 nm laser over the 633 nm laser. After the exposure of AgNP@R6G@BSA with A431 cells, the R6G spectrum could be clearly identified (Figure 5A). 

This proof-of-concept experiment demonstrates the possibility of using this AgNP@R6G@BSA nanosystem as a highly sensitive intracellular SERS label. Furthermore, the biocompatible BSA-covered surface in these AgNPs opens the possibility for further conjugation with recognition biomolecules (e.g., antibodies or DNA-BSA adducts) leading to different targeted delivery strategies.

In addition, to demonstrate an efficient internalization of the AgNP@R6G@BSA, A431 cells were labelled in order to identify the nuclei (Hoechst). Cells were then observed at the same time with a microscope both under fluorescence and dark field experimental settings. A perinuclear accumulation of AgNPs was clearly observed in merge images (Figure 5B).

**Figure 6:**
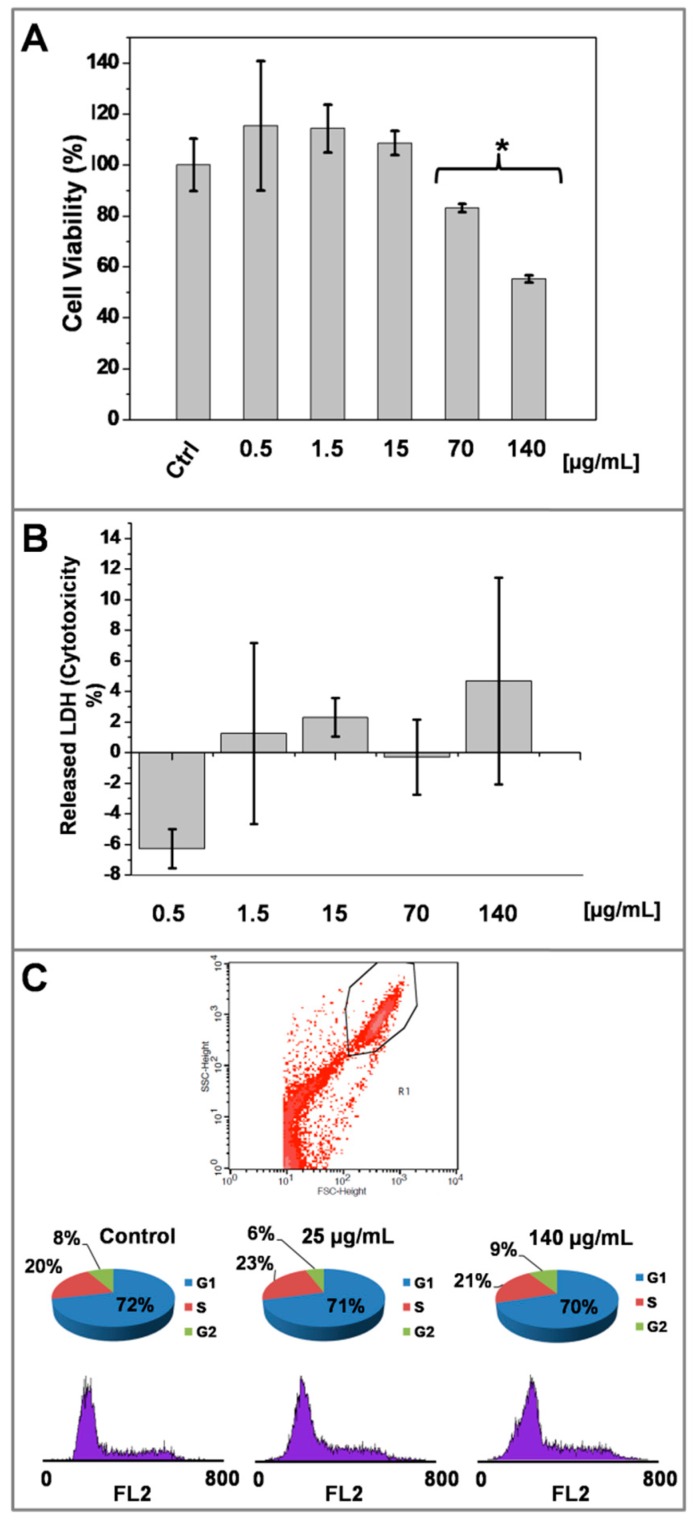
Effect of AgNP@R6G@BSA on A431 human epidermoid carcinoma cell line biocompatibility. Mitochondrial function (MTT reduction) after 24 h of treatment with increasing concentrations of AgNPs (**A**). Lactate dehydrogenase release assay (LDH) after 24 h of treatment with increasing concentrations of AgNP@R6G@BSA (**B**). Flow cytometry analysis of cell cycle after 24 h of treatment with increasing concentrations of AgNP@R6G@BSA (**C**). Data represented the mean ± SD. Measurements were made in three replicas for each experimental condition.

Key factors that influence further development of engineered nanoparticles for biological applications are firstly related to cellular biocompatibility. To this aim, we used three different experimental settings to assess cell metabolic activity, cell membrane integrity and cell cycle as readouts of cell viability after exposure of AgNP@R6G@BSA (Figure 6).

A431 human carcinoma cells exposure to increasing concentrations of AgNP@R6G@BSA from 0.3 μg/mL up to 140 μg/mL (referred also as ‘nanoparticle concentration’ farther ahead in the text) for 24 h was shown not to be detrimental to mitochondrial activity up to 70 μg/mL, (*p* < 0.05 U Mann-Whitney Test), as the MTT activity was maintained around or above 80% at all concentrations tested within this range. However, in the case of 140 μg/mL, cell viability decreased to 50%, implying an interference with the normal function of the mitochondria (Figure 6A). When we determined the extracellular LDH activity in A431 treated cells as a surrogate marker of cell death, mainly due to necrosis processes, we did not observe a substantial effect at any concentration tested (Figure 6B). The latter MTT reduction is inconsistent with the modest LDH values obtained in A431 cells treated at the highest concentration of AgNP@R6G@BSA, suggesting that AgNPs might drive apoptotic processes, characterized by a high degree of cell membrane integrity during the first 24 h.

The LDH results are in close agreement with the flow cytometry analysis obtained at the highest dose of nanoparticles. As shown in the scatter plot, A431 treated cells were not necrotic and the analysis of the subG1 cell cycle revealed no features of apoptosis, without differences respect to the untreated A431 cells (Figure 6C). Thus, AgNP@R6G@BSA seems to be compatible with key parameters related to cell viability at concentrations below 70 μg/mL.

## 4. Conclusions

Novel silver nanoparticles with elongated-shaped (AgNPs) and a size of about 13 ± 5 nm have been synthesized and characterized. With an SPR band in the near-IR zone (~800 nm) and BSA capping for biocompatibility, the newly synthesized AgNPs have potential uses as bioprobes, since living tissues do not absorb light energy in near-IR-zone.

The AgNP@R6G@BSA bioprobe has shown an extraordinary capacity to amplify the Raman signal of the R6G dye, reaching an AEF of 2.7 × 10^7^ at 633 nm and possibly even higher at 785 nm. This high AEF might be attributed to the joint contributions of the EM and CM components for the Raman signal enhancement as demonstrated in both the DFT calculations for the CM component and FEM calculations for the EM component. 

The AgNP@R6G@BSA bioprobes have been exposed to cells in culture, and demonstrated a very low toxicity, despite their high degree of internalization. A proof-of-concept experiment is also shown, in which an intracellular SERS spectrum is obtained. For future directions, we will use different NIR Raman dyes with this nanosystem platform and a wider variety of Raman-active dyes suitable for multiplex analysis. Additionally, detailed single-cell analysis of microRaman reconstructed imaging of our NPs might help to better understand the cell fate and internalization processes. Further antibody conjugation of the nanosystem will allow a more detailed molecular imaging of cancer cells.

## Supplementary Materials

The following are available online at http://www.mdpi.com/2079-4991/9/2/256/s1, Figure S1: Stability determinations by DLS in PBS (pH = 7.4) up to one month after synthesis procedure, Figure S2: Experimental and computational SERS spectra and main assignments for R6G, Table S1: Correspondence between the main peaks in the experimental and the calculated (DFT) spectra
