# Supplementary material for: Synthesis and Characterization of Elongated-Shaped Silver Nanoparticles as a Biocompatible Anisotropic SERS Probe for Intracellular Imaging: Theoretical Modeling and Experimental Verification
Source: Nanomaterials (Basel). 2019 Feb 13;9(2):256. doi: 10.3390/nano9020256 (PMC6409692; doi:10.3390/nano9020256)
Supplement: Supplementary file 1 [file nanomaterials-09-00256-s001.pdf]

Supporting Information

Synthesis and Characterization of Elongated-Shaped Silver Nanoparticles as a Biocompatible Anisotropic SERS Probe for Intracellular Imaging: Theoretical Modeling and Experimental Verification

Carlos Caro[1,2,3,4,*,†], Pedro Quaresma[5,†],Eulália Pereira[5], Jaime Franco[3,6], Manuel Pernia Leal [4,7], Maria Luisa García-Martín[4], Jose Luis Royo[8], Jose Maria Oliva-Montero[1], Patrick Jacques Merkling[1], Ana Paula Zaderenko[1], David Pozo[3,6,*] and Ricardo Franco[2,*]

[1] Department of Physical, Chemical and Natural Systems, Universidad Pablo de Olavide. Carretera de Utrera Km 1, 41013 Seville, Spain; josolimon@gmail.com (J.M.O.-M.); pjmerx@upo.es (P.J.M.)

[2] Departamento de Química, UCIBIO, REQUIMTE, Faculdade de Ciências, Universidade NOVA de Lisboa, 2829-516 Caparica, Portugal

[3] CABIMER, Andalusian Center for Molecular Biology and Regenerative Medicine, Av. Americo Vespucio, 24, 41092 Sevilla, Spain; jaime.munoz@cabimer.es

[4] BIONAND, Andalusian Centre for Nanomedicine and Biotechnology, Junta de Andalucía, Universidad de Málaga, Málaga, Spain; mpernia@us.es (M.P.L.); mlgarcia@bionand.es (M.L.G.-M.)

[5] Departamento de Química e Bioquímica, LAQV-REQUIMTE, Faculdade de Ciências, Universidade do Porto, 4169-007 Porto, Portugal; pedro.cq1@gmail.com (P.Q.); efpereir@fc.up.pt (E.P.)

[6] Department of Medical Biochemistry, Molecular Biology and Immunology, Universidad de Sevilla, Av. Sanchez Pizjuan, 4, 41009 Sevilla, Spain

[7] Department of Organic and Pharmaceutical Chemistry, Universidad de Sevilla, 41012 Seville, Spain

[8] Department of Biochemistry, Molecular Biology and Immunology, Universidad de Málaga, 29071 Málaga, Spain; joseluisroyo@uma.es

* Correspondence: ccaro@bionand.es (C.C.); david.pozo@cabimer.es (D.P.); rft@fct.unl.pt (R.F.); Tel.: +34-954-467-841 (D.P.)

† These authors contributed equally to this work.

To ensure the stability of the NPs, the DLS have been plotted: just after the synthesis and 1 month after.

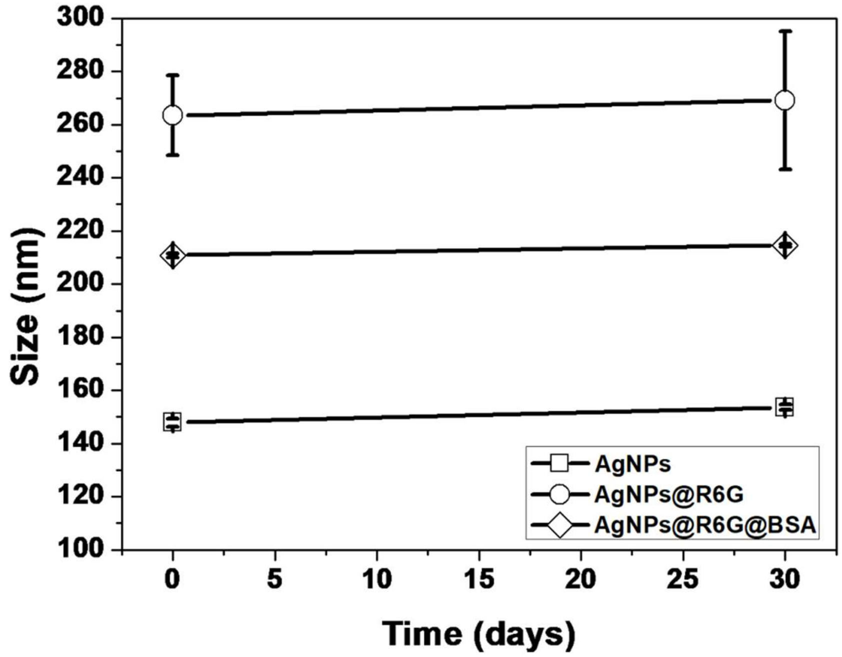

Figure S1. Hydrodynamic diameters of AgNPs (squares), AgNPs@R6G (spheres) and AgNPs@R6G@BSA (diamond)

In order to compare different experimental settings and some calculated data, the spectra of pure R6G, AgNP@R6G@BSA and calculated DFT interaction between R6G and silver atoms are shown:

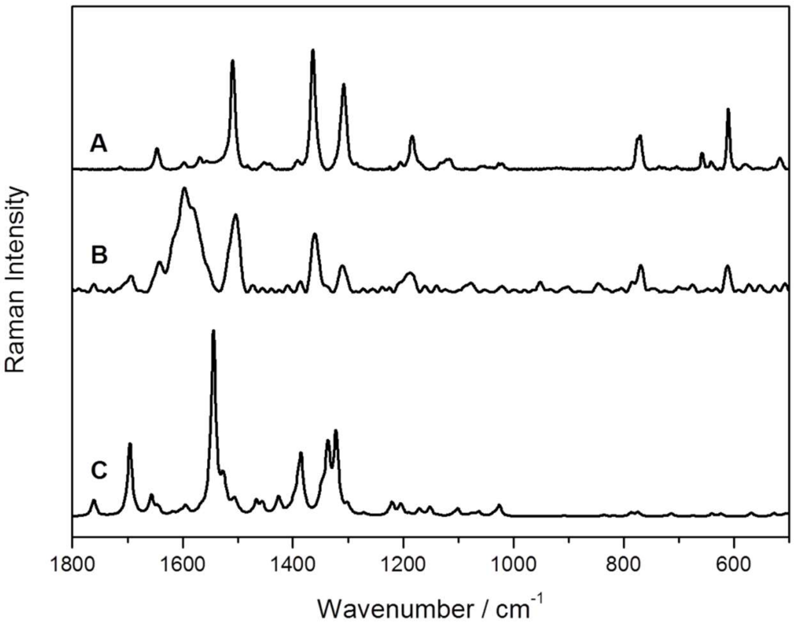

Figure S2. Comparison of Experimental Raman spectrum of pure R6G (A), Experimental SERS spectrum of AgNP@R6G@BSA (B) SERS spectrum obtained by DFT calculation (R6G interacting with a thin layer of 10 atoms of Ag) (C).

Table S1 shows the correspondence between the main peaks in the experimental and the calculated (DFT) spectra. All the assignments are related to Aromatic C–C stretching.

**Experimental Wavenumber (cm^{-1})**	**Computational Wavenumber (cm^{-1})**
**1646**	**1695**
**1509**	**1544**
**1452**	**1464**
**1363**	**1386**
**1307**	**1335**
**1307**	**1322**

Table S1. Comparison of changes in wavenumbers between the experimental Raman spectrum and the computational Raman spectrum.
